# Determination of β_2_-Agonist Residues in Fermented Ham Using UHPLC-MS/MS after Enzymatic Digestion and Sulfonic Resin Solid Phase Purification

**DOI:** 10.3390/molecules28052039

**Published:** 2023-02-21

**Authors:** Chenggang Cai, Yannan Xiang, Siyi Tian, Zhongce Hu, Zhengyan Hu, Bingjie Ma, Pinggu Wu

**Affiliations:** 1 College of Biological and Chemical Engineering, Zhejiang University of Science and Technology, Hangzhou 310023, China; 2 College of Biotechnology and Bioengineering, Zhejiang University of Technology, Hangzhou 310014, China; 3 Zhejiang Provincial Center for Disease Control and Prevention, Hangzhou 310051, China

**Keywords:** matrix effects, β_2_-agonist residues, ham, solid phase extraction, UHPLC-MS/MS

## Abstract

β_2_-agonists are a class of synthetic sympathomimetic drugs with acute poisoning effects if consumed as residues in foods. To improve the efficiency of sample preparation and to overcome matrix-dependent signal suppression in the quantitative analysis of four β_2_-agonists (clenbuterol, ractopamine, salbutamol, and terbutaline) residues in fermented ham, an enzyme digestion coupled cation exchange purification method for sample preparation was established using ultra-high performance liquid chromatography and tandem mass spectrometry (UHPLC-MS/MS). Enzymatic digests were subject to cleanup treatment on three different solid phase extraction (SPE) columns and a polymer-based strong cation resin (SCR) cartridge containing sulfonic resin was found to be optimal compared with silica-based sulfonic acid and polymer sulfonic acid resins based SPEs. The analytes were investigated over the linear range of 0.5 to 10.0 μg/kg with recovery rates of 76.0–102.0%, and a relative standard deviation of 1.8–13.3% (n = 6). The limit of detection (LOD) and the limit of quantification (LOQ) were 0.1 μg/kg and 0.3 μg/kg, respectively. This newly developed method was applied to the detection of β_2_-agonist residues in 50 commercial ham products and only one sample was found to contain β_2_-agonist residues (clenbuterol at 15.2 µg/kg).

## 1. Introduction

**Figure 1:**
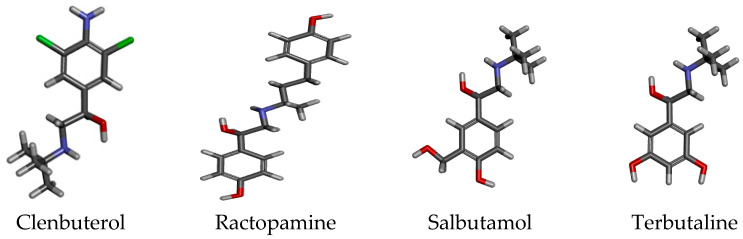
Structures of the four β_2_-agonists. Red is O, blue is N, green Cl, black C, grey H.

β_2_-Agonists, such as ractopamine, clenbuterol, terbutaline, and salbutamol (Figure 1), are a class of synthetic sympathomimetic drugs that have been used in the clinical treatment of asthma for many years [1]. Because of their effects on carcass repartition, these compounds have also been used as growth-promoting drugs for cattle, sheep, and swine, leading to the presence of β_2_-agonist residues in the resulting meat products [2,3]. It has been proven that the consumption of meat that contains β_2_-agonist residues is able to cause acute poisoning of headache, tachycardia nausea, dizziness, and others [4,5,6]. Thus, β_2_-agonists have been prohibited for use as feed additives in China and must not be detectable in animal product foods, especially ractopamine, clenbuterol, terbutaline, and salbutamol, which are the are mainly monitored varieties of poultry products in China. Even so, abuse of these compounds has occurred in recent years, which has resulted in serious food safety problems for consumers. It is very important to detect the residues in food materials that probably used β_2_-agonists, such as animal foods materials.

There were some different analytical methods of gas chromatography–mass spectrometry (GC-MS) [7,8], enzyme-linked immunosorbent assay (ELISA) [9], liquid chromatography–tandem mass spectrometry (LC-MS/MS) [10,11,12], capillary electrophoresis mass-spectrometry (CE-MS) [13], and ultra-high performance liquid chromatography–tandem mass spectrometry (UHPLC-MS/MS) [12,14,15,16,17,18,19] that have been utilized for detecting β_2_-agonists in food material or food samples, such as pork meat, liver, and feed [17,19,20]. Among the analyzed methods, LC–MS/MS and UHPLC–MS/MS are the main utilized methods for β_2_-agonist residues analysis because of their outstanding accuracy, sensitivity, and reproducibility. During the pretreatment procedures, the samples were usually processed by enzymatic or acid-mediated digestion treatments for the extraction of the β_2_-agonists chemicals, then the liquefied samples were further purified by liquid-liquid extraction (LLE) or solid phase extraction (SPE). The commonly used matrix in SPE includes silica-based sulfonic acid and polymer sulfonic acid resins, other resins such as sulfonic resin had rarely been used in SPE for β_2_-agonists chemicals purification.

There were few reports on the analysis of β_2_-agonist residues in processed meats such as ham [21,22,23]. Ham is a kind of fermented pork product, the ham matrix is more complicated than that of fresh meat material, there were chemicals from the pork material and metabolites from microbes during the fermentation process. In addition, the quantitative analysis of β_2_-agonist residues in ham by UHPLC–MS/MS will be affected by the matrix effect, resulting in high signal deviations. Therefore, sample preparation of sample shows a critical role in the accurate analysis of β_2_-agonist residues in ham. While LLE is a classically employed extraction method, emulsification occurs frequently and adds a level of difficulty in the sample preparation procedure [17,24]. Sample purification by SPE directly without a preparatory LLE step would greatly simplify and accelerate the sample preparation process. Despite SPE finding frequent application in the detection of β2-agonist residues in foodstuffs and feed, the ham matrix has yet to be studied in detail [23,25].

The aim of this study was to establish a simple and effective method for sample purification prior to UHPLC-MS/MS detection of β_2_-agonists in ham. The ham samples were prepared by enzymatic degradation and SPE-mediated cleanup without a preparatory LLE step. For SPE, strong cation exchange matrices were investigated, and four typical β_2_-agonists were selected as model analytes.

## 2. Results

### 2.1. Sample Preparation Optimization

The selection of a suitable cartridge is critical for SPE. While studies have reported using C18 [26], MCX (*N*-vinyl-2-pyrrolidone–divinylbenzene polymer sulfonic acid resin) [18,23], and SCX (silica-based sulfonic acid resin) [12] SPE cartridges for analysis of β_2_-agonist residues in foodstuff and feed, as for the fermented ham product, there was limited research reported using SPE for cleanup of ham samples. As one of the special meat products, the ham samples were stored at special conditions for a period of time called fermentation. Many ingredients will change during the long-term storage, for example, the degradation of protein to amino acids, lipid oxidation, the changes in enzymes from microorganisms responsible for biogenic amines production, and some flavors created during the fermentation process, especially the biogenic amines, which were the mainly interfering substances. It is important to select the appropriate SPE cartridge for the cleanup of ham samples because of the interference components presented, which will influence the analysis results. In this study, the four representative β_2_-agonist compounds (2.0 μg/kg) were spiked into the blank ham, and the results from the standard recovery tests are presented in Table 1. Clenbuterol was recovered in high yields from all three test kinds of SCX, MCX, and SCR (polystyrene–divinylbenzene polymer sulfonic acid resin) SPE cartridges. Therefore, all three cation exchange cartridges were suitable for clenbuterol analysis. However, terbutaline recovery from SPE ranged between 42.7% to 83.0%. Recovery percentages for ractopamine and salbutamol were between that of the clenbuterol and terbutaline extremes. The recovery ranges for the four β_2_-agonist from SCX, MCX, and SCR were 42.7–95.2%, 62.6–97.4%, and 83.0–98.0%, respectively. For all four β_2_-agonist compounds, the SCR cartridge provided the best recovery percentages and was selected for the next experiments.

### 2.2. Chromatographic Conditions Optimization

In order to achieve the ideal sensitivity, separation, running time, and symmetrical peak shape of the four analytes and internal standard, the separation column and mobile phase were optimized.

An Acquity Waters BEH C18 column (100 mm × 2.1 mm, 1.7 μm) was used with a satisfactory separation result. The 5 mM aqueous ammonium acetate and acetonitrile (with 1% formic acid) as mobile phases resulted in a very good selectivity. The retention time and ions monitored for the representative β_2_-agonists and their isotopically labeled internal standards are listed in Table 2. The internal standard for clenbuterol and ractopamine were D_9_-clenbuterol and D_3_-ractopamine, respectively, and D_6_-salbutamol was used as the internal standards for both salbutamol and terbutaline.

### 2.3. Calibration Curves

The linear weighted correction curve was established according to the peak area ratio of the compound to the corresponding internal standard. The calibration range was from 0.2 to 5 μg/L, and the calibration curves were evaluated by a six times analysis with one replicate at each calibration point. The correlation coefficients of the calibration curves of each analyte ranged from 0.995 to 1.000. Table 3 showed the linear range, regression equation, and correlation coefficient. Linearity was assessed by the deviation of the average calculated concentration over three tests, which remained within ±15% of the nominal concentration and the coefficient of variation (CV) < 15%. Impurities had no significant effect on the quantification of analytes.

### 2.4. Method Validation

A method for analysis of β_2_-agonist residues in hams by solid phase extraction using an SCR cartridge coupled with UHPLC-MS/MS was developed. The selectivity, sensitivity, linearity, precision, accuracy, and recovery of the method were validated in this study. The results were as follows.

### 2.5. Precision and Accuracy

The precision and accuracy of the method were obtained by analyzing the QC points of each compound. The accuracy and precision data are shown in Table 4. The intra-assay accuracy of clenbuterol, ractopamine, and salbutamol was 83.2%–107.0%, and that of terbutaline was 73.9%–90.8%. The RSDs for the intra-day precision were between 1.8% and 13.3%. The inter-day accuracy and precision showed good reproducibility for all six measurements with an accuracy that ranged from 76% to 102%, and RSDs for the intra-day precision varied from 2.6% to 12.8%.

### 2.6. Assessment of the Matrix Effect

The peak areas of the analytes added to the blank ham sample were compared with the ham-free methanol solution prepared with the same amount of the analyte of interest. The standards isotopically labeled made the quantification more accurate. D_6_-salbutamol was used as an internal standard for terbutaline. As shown in Table 3, a minor suppression-type matrix effect (where ME < 1) was observed. The ME values for clenbuterol, ractopamine, salbutamol, and terbutaline varied from 65.4% to 92.4%, indicating that the matrix effect was not significant for clenbuterol, ractopamine, and salbutamol.

### 2.7. Selectivity and Sensitivity

**Figure 2:**
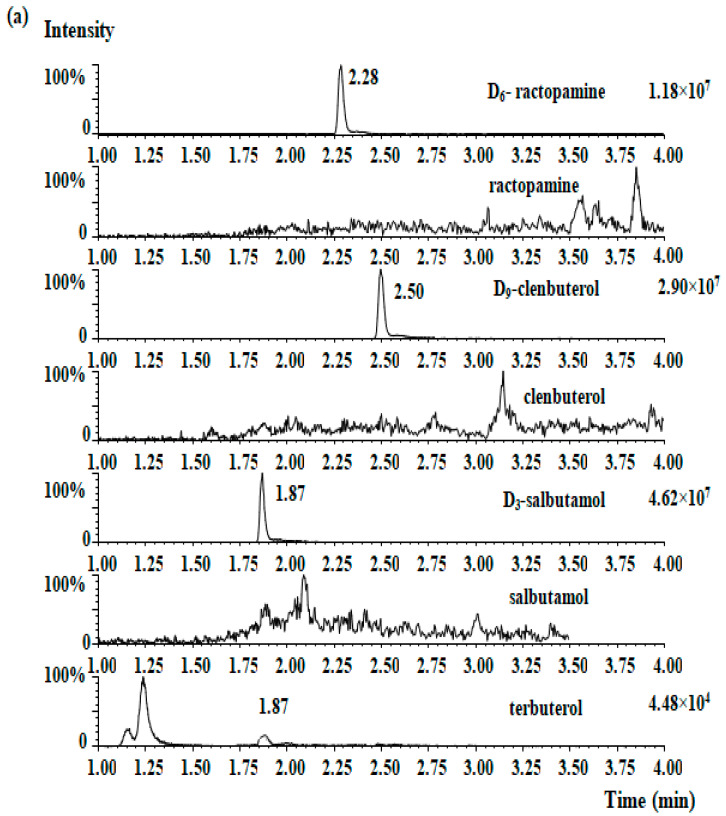

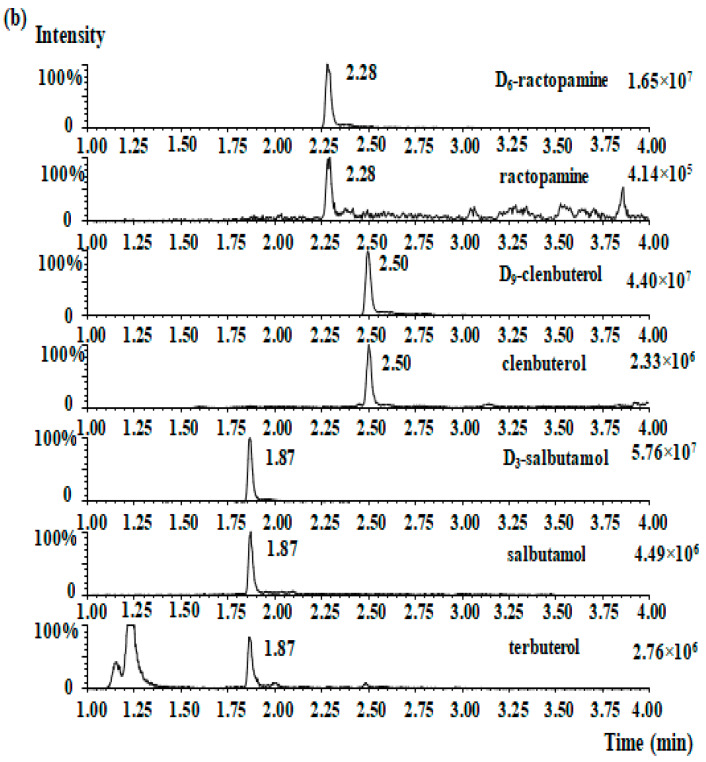

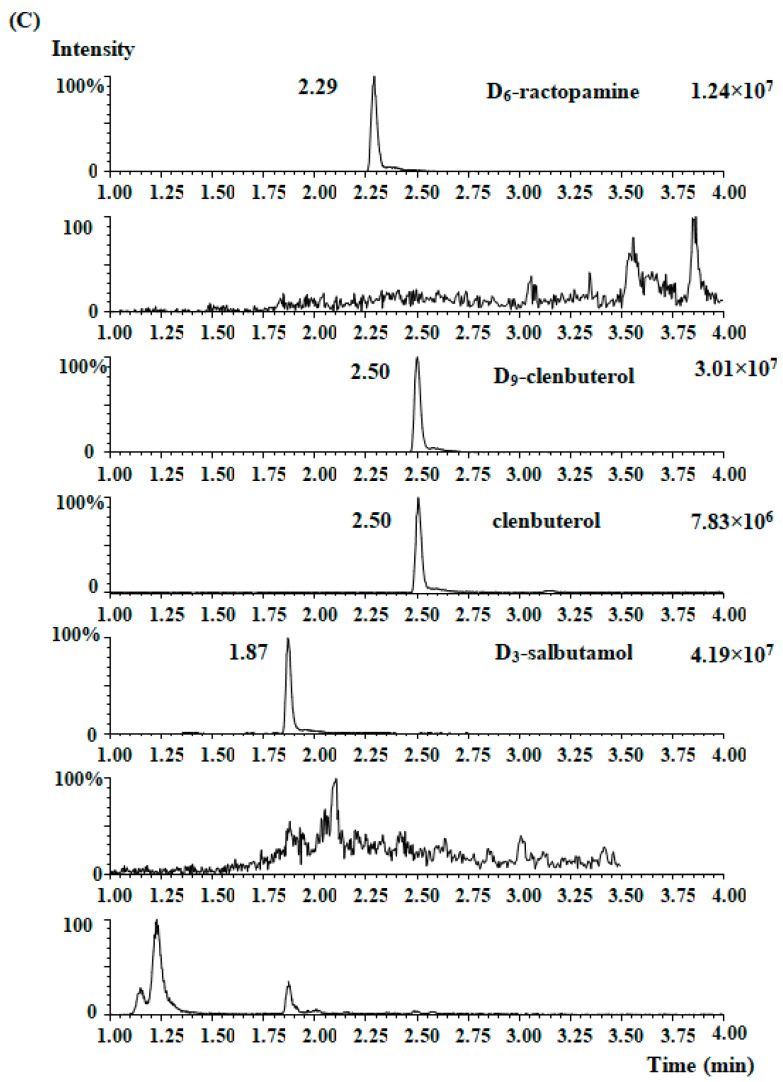
MRM chromatographs of four β_2_-agonists in the blank ham sample (**a**), the blank spiked ham sample (**b**), and the presence of clenbuterol (15.2 µg/kg) in one ham sample (**c**).

The selectivity was determined by comparing the chromatogram of β_2_-agonist-free ham and β_2_-agonists-spiked ham. Within the four standards retention times, there were no interfering compounds peaks. The MRM chromatograms of the four β_2_-agonists for blank ham samples and samples that were spiked at 2.0 μg/kg are shown in Figure 2a,b, respectively. The LOD of each analyte was 0.1 µg/kg and the LOQ of each analyte was 0.3 µg/kg.

### 2.8. Real Samples Analysis

The established method was then employed to detect the presence of β_2_-agonist residues in fermented hams. Fifty real samples of Chinese traditional ham (assorted brands) were collected for clenbuterol, ractopamine, salbutamol, and terbutaline analysis. Except for one ham sample, in which clenbuterol was detected (15.2 µg/kg, Table 5), none of the four β_2_-agonists were detected in the other forty-nine samples, it can be speculated that clenbuterol is still the mostly used β_2_ agonist in China. Figure 2c shows the MRM chromatogram of the positive sample. There was no interference in the chromatogram indicating a quantitative or qualitative transition.

## 3. Discussion

There are some studies investigating the presence of β_2_-agonist residues in solid samples, such as pork, liver, and feed [17,19,20], and few studies report on the analysis of β_2_-agonist residues in complicated matrices such as ham [21,22,23]. Ham, which is a type of fermented meat, is very dry and hypersalinated because it is manufactured over several months by salting, curing, and drying. It contains many co-eluting interfering degraded substances, such as amides, peptides, N-nitrosamines, and especially, biogenic amines, which are aliphatic, aromatic, or heterocyclic organic bases, such as histamine, cadaverine, putrescine, 2-phenylethylamine, tyramine, spermine, tryptamine and spermidine [27]. These biogenic amines have a similar chemical structure to β_2_-agonists, and β_2_-agonists may be biodegraded to other complex and unclear chemicals by enzymes and microorganisms during fermentation and storage, which results in problematic sample separation and purification, collectively referred to as matrix effects. While matrix effects are a recognized hurdle in the analysis of β_2_-agonist residues in ham, few studies have tackled this challenge [23].

The matrix effect was the difference between the mass spectral response of the analyte in a standard solution and that of the same analyte in the matrix. Matrix composition may change the target analyte ionization efficiency, thus affecting the efficiency and reproducibility of the quantitative methods and the accuracy and sensitivity of LC-MS/MS methods [28]. To decrease the matrix effect, the samples must be “cleaned up”; that is, the analytes of interest need to be extracted from the samples “cleanly”. For sample cleanup, past studies have employed strong cation exchange cartridges for SPE prior to UHPLC–MS/MS analysis [23]. In this study, MCX, SCX, and SCR SPE cartridges, sulfonic acid cation exchange cartridges employing *N*-vinyl-2-pyrrolidone–divinylbenzene, silica gel, and polystyrene–divinylbenzene matrices were employed, respectively [19,23,29]. MCX, SCX, and SCR cartridges were suitable for ham sample cleanup, and the SCR was the optimal one. The core matrices in the SCX and MCX cartridges are silica gel and a mixed-mode polymeric sorbent, respectively. Silica gel leads to strong non-specific adsorption of biological macromolecules, especially peptides and proteins. MCX, which is used widely in β_2_-agonists analysis, enables both ion exchange and reverse-phase adsorption; it can especially absorb and separate alkaline compounds. Strong cation exchange cartridges strongly adsorb terbutaline, which results in difficulties in elution during SPE and low recovery of this analyte. Thus, terbutaline provides for a more difficult quantification than the other β_2_-agonists.

The MCX and SCX cartridges were eluted with 5% ammoniated methanol. The SCR cartridge was eluted with 5% ammoniated ethyl acetate (10 mL). Relatively, 5% ammoniated methanol as the eluent solvent will obtain eluent with more polarity than the eluent by 5% ammoniated ethyl acetate, and it will bring more interference during analysis [30].

An analytical method for the detection of salbutamol in ham sausage using molecularly imprinted solid phase extraction (MISPE) coupled with HPLC has been reported, MISPE shows application potential for selective extraction or clean-up of target analytes from different complex matrices, however, only one β_2_-agonist residue could be analyzed, and scope of this method was limited [21]. The LOD and LOQ in this work were lower than that reported by Lu et al. [22]. A comparison of the newly developed method to previously reported methods is presented in Table 6. In conclusion, a simple and highly effective sample cleanup was achieved for the simultaneous determination of the four β_2_-agonist residues in fermented ham.

## 4. Materials and Methods

### 4.1. Materials and Samples

Clenbuterol hydrochloride (purity: 98.5%), ractopamine hydrochloride (purity: 97.0%), salbutamol (purity: 99.5%), terbutaline (purity: 98.5%), D_9_-clenbuterol (purity: 98.0%), D_3_-ractopamine(purity: 98.0%), and D_6_-salbutamol (purity: 98.0%) standards were obtained from Sigma (St. Louis, MO, USA). Methanol (HPLC grade), acetonitrile, and ethyl acetate were obtained from DIMA Technology Inc. (Richmond, VA, USA). The MCX (*N*-vinyl-2-pyrrolidone–divinylbenzene polymer sulfonic acid resin) and SCX (silica-based sulfonic acid resin) SPE cartridges were obtained from Waters (Milford, MA, USA, 500 mg/6 mL). The SCR (polystyrene–divinylbenzene polymer sulfonic acid resin, 500 mg/6 mL) SPE cartridges were obtained from J&K Scientific (Beijing, China). Ultra-pure water was obtained using a Milli-Q reverse osmosis water polishing system (Millipore, Milford, MA, USA). Stock solutions (1.0 mg/mL) of clenbuterol, ractopamine, salbutamol, and terbutaline in methanol were prepared and stored at −18 °C. Working solutions of each compound at appropriate concentrations for calibration curves establishment and spiking tests were prepared from stock solutions using methanol immediately prior to use.

A sample of traditional Chinese ham, which is a type of dry-cured ham aged for 8 to 10 months, was purchased from a local supermarket (Wal-Mart Stores, Inc. Hangzhou, China). Another ham bought from Hangzhou Lianhua Supermarket was confirmed to be free of β_2_-agonist residues by SPE coupled with UHPLC-MS/MS using existing methods and named “blank ham”, the hams were stored at −18 °C for less than one year prior to analysis. Samples for quality control (QC) were prepared by spiking standard β_2_-agonists stock solutions into blank ham samples.

### 4.2. Sample Preparation

The skin of the sample ham was removed, and the sample was cut up and homogenized in a dispersive homogenizer. Homogenized sample (2.0 g) was weighed and transferred into a 50 mL centrifuge tube (50 mL) and spiked with internal standard solutions (40 μL) containing D_9_-clenbuterol, D_3_-ractopamine, and D_6_-salbutamol (250 μg/L). Then, 25 mL of sodium acetate buffer solution (0.2 mol/L, pH 5.2) was added to the tube. The mixture was homogenized for 30 s and incubated with 100 μL of β-glucuronidase/arylsulfatase (CFEQ-4-150001-0002, ANPEL Scientific Instrument Co., Ltd., Shanghai, China) in a water bath (37 °C) for 16 h and then cooled to room temperature. The mixture was then centrifuged at 12,000× *g* for 10 min at 4 °C, and the pH of the transferred supernatant was adjusted to 2.0 ± 0.1 by using 3.0 M HCl. This mixture was centrifuged again (12,000× *g*, 10 min, 4 °C) and the enzymatic hydrolysate supernatant was further purified by SPE.

### 4.3. Sample Purification

SPE was carried out on the enzymatic hydrolysate using MCX, SCX, and SCR cartridges. Each SPE cartridge was conditioned sequentially with water (5 mL), methanol (5 mL), water (5 mL), and HCl (5 mL, 40 mM). Enzymatic hydrolysate (10 mL) was loaded onto the cartridge, and the cartridge was washed with water (5 mL) and methanol (5 mL). The SCR cartridge was brought to dryness by action of a vacuum pump and then eluted with 5% ammoniated ethyl acetate (10 mL). The MCX and SCX cartridges were eluted with 5% ammoniated methanol (10 mL). The eluates were nitrogen dried in a 40 °C water bath. The residue was re-dissolved in 1 mL of methanol–water solution (10%, *v*/*v*), after filtering through a 0.22 μm organic filter, the eluate was analyzed by UHPLC-MS/MS.

### 4.4. UHPLC-MS/MS System

The UHPLC instrument (Waters Corp., Milford, MA, USA) with a BEH C18 column (100 mm × 2.1 mm, 1.7 μm) was used. The gradient elution at 0.3 mL/min using mobile phase (A): 5 mM aqueous ammonium acetate, and mobile phase (B): 0.1% formic acid in acetonitrile were carried out at 40 ℃ with the injection volume of 2 μL. The chromatographic conditions were 95% A and 5%B (0 to 0.5 min); 50% A and 50% B (0.5 to 2.0 min); 5% A and 95% B (2.0 to 5.5 min); 95% and 5% B (5.5 to 6.0 min).

An electrospray ionization interface was used to connect a Xevo TQS triple quadrupole mass spectrometer (Waters Corp., Milford, MA, USA) and LC system. A positive ionization mode was performed for β_2_-agonists analysis. The ionization source was 150 °C and 3 kV capillary voltage, the cone gas was 60 L/h, and desolvation at 400 °C with desolvation gas of 500 L/h. The multiplier was 650 V. The cone and collision energy were optimized, and the data collection was performed in multiple reaction monitoring (MRM) modes. Table 1 presents the optimal parameters and MRM transitions used to determine and quantify the four β_2_-agonists. The MRM parameters for the optimum yield product ions of the internal standard and each chemical eluted from the LC column were defined. The MassLynx software (Version 4.1) was used for data processing. The analysis time includes the time of sample preparation, purification, and analysis. the enzyme digestion was set as 16 h, and the purification and LC-MS analysis need about 2 h, so the whole time was about 18 h.

### 4.5. Validation of Method

#### 4.5.1. Linearity Analysis

Five standard solutions of the four compounds were prepared and the results were analyzed after three independent analyses, while data fitting was performed using a linear regression based on the ratio of the peak area of the analyte to the peak area of the corresponding internal standard. The linearity was evaluated with the control samples at different levels over 6 days.

#### 4.5.2. Accuracy, Precision and Recovery

Three different standard levels (covering the calibration curve range) were spiked into QC samples for analysis of precision and accuracy. Six times were performed for every sample. After calculating the within-day and inter-day results, the relative standard deviation (RSD) and the repeatability of the method were analyzed. The accuracy of the method was defined as the percentage deviation between the measured and nominal concentration. The values of recovery were calculated by comparing the β_2_-agonist concentrations added to the ham samples with the ham sample concentrations analyzed from the calibration curve. The criteria for the method precision and accuracy were assessed as follows: the RSD value was less than 20% for the limit of quantification (LOQ), and at each concentration level, the RSD value determined should be less than 15%. In addition, the recovery value should be within 70% to 120%.

#### 4.5.3. Matrix Effect

If defined the analyzed standard solutions peak areas is A, the corresponding extracted standards spiked into ham samples peak areas was B, and the matrix effect (ME) values can be calculated as follows [19,31]:

ME (%) = B/A × 100(1)

A value of 100% indicates that no matrix effect is observed and the response in the mobile phase and ham extracts are the same. A value above and below 100% indicates an ionization enhancement and suppression, respectively.

#### 4.5.4. Limit of Detection and Limit of Quantification

The limit of detection (LOD) was set to a minimum concentration 3 times higher than the background noise (S/N > 3). The limit of quantification (LOQ) was selected as the lowest concentration of fermented meat that could be determined in the experiment when the signal-to-noise ratio exceeded 10.

#### 4.5.5. Specificity

The method specificity was assessed by evaluating potential interference from other components. Ham free from β_2_-agonist residues and ham containing β_2_-agonist residue were spiked with known concentrations of the four β_2_-agonist residues and processed for analysis according to the method developed in this study. The specificity was obtained by comparing these two chromatograms.

#### 4.5.6. Statistical Analysis

Significant differences in mean levels of clenbuterol, ractopamine, salbutamol, and terbutaline in samples were identified by one-way analysis of variance (ANOVA) and least significant difference (LSD) test at *p* < 0.05. Analysis was conducted using the SPSS 20.0 software package (SPSS Inc., Chicago, IL, USA).

## 5. Conclusions

An effective analytical method for the simultaneous analysis of four β_2_-agonist residues (clenbuterol, ractopamine, salbutamol, and terbutaline) in fermented ham was developed. Samples were processed with an enzymatic digestion step followed by SPE cleanup. Several SPE cartridges were investigated for sample cleanup and the SCR column, a sulfonic acid cation exchange column supported on polystyrene–divinylbenzene, was found to be the most effective general cartridge for β_2_-agonist residue recovery. After SPE cleanup, the obtained extracts were subjected to UHPLC-MS/MS analysis (LOD = 0.1 μg/kg, LOQ = 0.3 μg/kg). This newly developed method was applied to the detection of β_2_-agonist residues in 50 commercial ham products and only one sample was found to contain β_2_-agonist residues (clenbuterol at 15.2 µg/kg).

## Figures and Tables

**Table 1:** SPE recovery results.

Cartridge	Clenbuterol Recovery RSD		Ractopamine Recovery RSD		Salbutamol Recovery RSD		Terbutaline Recovery RSD	
	(%)	(%)	(%)	(%)	(%)	(%)	(%)	(%)
SCX	95.2	8.5	78.6	8.9	63.4	10.5	42.7	12.8
MCX	97.4	7.5	94.3	9.8	82.6	7.2	62.6	11.6
SCR	98.0	4.3	94.5	7.8	91.3	5.4	83.0	10.2

**Table 2:** Mass spectrometry parameters for the determination of β_2_-agonists.

Compound	Internal Standard	Parent (*m/z*)	Daughter (*m/z*)	Cone Voltage (v)	Collision Energy (v)	Retention Time (min)
Clenbuterol	D_9_-clenbuterol	277.29	203.11 */259.0	14	16/16	2.50
Ractopamine	D_3_-ractopamine	302.35	164.23 */284.0	44	16/16	2.29
Salbutamol	D_6_-salbutamol	240.35	148.16 */222.0	6	18/12	1.87
Terbutaline	D_6_-salbutamol	226.22	152.19 */125.0	4	18/18	1.87
D_9_-clenbuterol		286.35	204.13 *	4	16	2.50
D_3_-ractopamine		308.42	168.21 *	10	16	2.29
D_6_-salbutamol		243.35	151.16 *	4	18	1.87

Note: * is the quantification ion.

**Table 3:** Calibration data for the four β_2_-agonists: regression equation, correlation coefficient, LOD, LOQ, matrix effect, and internal standard used for each compound.

Compound	Regression Equation	Correlation Coefficient (r2)	LODs (μg/kg)	OQs (μg/kg)	Matrix Effect (%)	Internal Standard
Clenbuterol	y = 0.95x − 0.02	1.000	0.1	0.3	92.4	D_9_-clenbuterol
Ractopamine	y = 1.92x − 0.04	0.998	0.1	0.3	86.7	D_3_-ractopamine
Salbutamol	y = 12.15x − 0.30	0.999	0.1	0.3	74.3	D_6_-salbutamol
Terbutaline	y = 0.42x + 0.02	0.997	0.1	0.3	65.4	D_6_-salbutamol

**Table 4:** Accuracy and precision for the determination of β_2_-agonists in ham using an SCR SPE cartridge (n = 6).

Target Standard	Spiked Levels (μg/kg)	Precision (RSD, %)		Accuracy (Recovery, %)	
		Intra-Day	Inter-Day	Intra-Day	Inter-Day
Clenbuterol	0.5	8.5	10.2	92.6	90.2
	2.0	4.3	6.9	98.0	96.5
	10.0	1.8	4.6	102.0	102.0
Ractopamine	0.5	9.4	9.6	89.0	89.6
	2.0	7.8	8.7	94.5	93.0
	10.0	3.4	4.2	107.0	95.6
Salbutamol	0.5	10.4	10.4	83.2	86.2
	2.0	5.4	8.0	91.0	89.5
	10.0	3.2	2.6	96.2	93.8
Terbutaline	0.5	13.3	12.8	73.9	76.0
	2.0	10.2	6.3	83.0	78.0
	10.0	8.6	4.6	90.8	86.2

**Table 5:** Clenbuterol contents in the ham (µg/kg).

Analysis Times	Sample Weight (g)	PEAR Area	Clenbuterol Content	Mean Value + Standard Error
1	2.0	7.83 × 10^6^	15.2	
2	2.0	7.67 × 10^6^	14.9	15.2 ± 0.2
3	2.0	7.98 × 10^6^	15.5	

**Table 6:** Comparison between proposed method and the previous literature.

NO	Matrix	Pre-Treatment Procedure	The Number of β_2_-Agonist	Instrument	LOD (μg/kg)	LOQ (μg/kg)	Reference
1	Sausage	Enzymatic digestion, MSPD	4	HPLC-MS/MS	0.1–0.15	0.25–0.40	[22]
2	Ham sausage	LLE, MISPE	1	HPLC	0.2	0.68	[21]
3	Pork, sausage, and milk powder	Acid digestion, on line SPE	3	LC-MS/MS	0.0073–0.088	0.024–0.29	[23]
4	Pork, beef, mutton, and chicken	Enzymatic digestion, LLE, SPE	12	UHPLC-Q-Orbitrap HRMS	0.0033–0.01	0.024–0.29	[17]
5	Beef muscle, beef liver, goat muscle, and goat liver	Enzymatic digestion, QuEChERS	10	LC-MS/MS	0.2–0.9	0.8–3.2	[20]
6	Porcine muscle	Improved QuEChERS	14	UHPLC-Q-Orbitrap MS	0.17–1.67	0.56–5.00	[19]
7	Ham	Enzymatic hydrolysis, SPE	4	UHPLC-MS/MS	0.1	0.3	this work

## Data Availability

All data are in the manuscript.

## Sample Availability

Samples are available from the authors.
